# Deep-Sea Natural Products from Extreme Environments: Cold Seeps and Hydrothermal Vents

**DOI:** 10.3390/md20060404

**Published:** 2022-06-19

**Authors:** Mengjing Cong, Xiaoyan Pang, Kai Zhao, Yue Song, Yonghong Liu, Junfeng Wang

**Affiliations:** 1 CAS Key Laboratory of Tropical Marine Bio-Resources and Ecology, Guangdong Key Laboratory of Marine Materia Medica, Innovation Academy of South China Sea Ecology and Environmental Engineering, South China Sea Institute of Oceanology, Chinese Academy of Sciences, Guangzhou 510301, China; congmengjing21@mails.ucas.ac.cn (M.C.); xypang@scsio.ac.cn (X.P.); zhaokai04222@163.com (K.Z.); songyue202205@163.com (Y.S.); yonghongliu@scsio.ac.cn (Y.L.); 2 University of Chinese Academy of Sciences, 19 Yuquan Road, Beijing 100049, China; 3 Southern Marine Science and Engineering Guangdong Laboratory (Guangzhou), Guangzhou 511458, China

**Keywords:** natural products, extreme environments, cold seeps, hydrothermal vents, bioactivities

## Abstract

The deep sea has been proven to be a great treasure for structurally unique and biologically active natural products in the last two decades. Cold seeps and hydrothermal vents, as typical representatives of deep-sea extreme environments, have attracted more and more attention. This review mainly summarizes the natural products of marine animals, marine fungi, and marine bacteria derived from deep-sea cold seeps and hydrothermal vents as well as their biological activities. In general, there were 182 compounds reported, citing 132 references and covering the literature from the first report in 1984 up to March 2022. The sources of the compounds are represented by the genera *Aspergillus* sp., *Penicillium* sp., *Streptomyces* sp., and so on. It is worth mentioning that 90 of the 182 compounds are new and that almost 60% of the reported structures exhibited diverse bioactivities, which became attractive targets for relevant organic synthetic and biosynthetic studies.

## 1. Introduction

Extreme environments refer to areas close to the limits of life, such as cold seeps, hydrothermal vents, polar and hot regions, or marine areas with high salinity [1]. Because of the extreme conditions of pressure, temperature, or high concentrations of toxic elements, unique organisms are more likely to appear. Compared with other ecosystems, extreme environments have not been fully developed and utilized, due to the limited conditions and difficult sampling. In recent years, with the progress of technology and the further exploration of the deep sea, scientists have gradually realized the uniqueness of natural products from extreme environments [2].

Cold seeps are typical deep-sea, chemosynthetically driven ecosystems, characterized by methane-rich fluid emissions and distinctive sulfur oxidation–reduction reactions, which lead to a high abundance of specialized cold-seep microorganisms [3]. The temperature of cold seeps is 2–4 °C, basically the same as the temperature around the seafloor. Microorganisms and animals from deep-sea cold seeps, which could be a new source of biomedically important compounds, due to their unique habitat, are only beginning to be investigated. The great potential for natural product discovery in deep-sea cold seep organisms will undoubtedly accelerate the investigation of new drugs [4].

Hydrothermal vents are formed when water heated in the Earth’s crust and magma are forced explosively to the surface through rock fissures in volcanic regions. Since ocean hydrothermal vents are among the most dynamic environments on Earth, secondary metabolite diversity of this extreme environment is considerably high [5]. With advances in sample collecting techniques, deep-sea hydrothermal vents might be potential hot spots for natural product discovery [6].

Therefore, this review covers papers on metabolites isolated from deep-sea extreme environments, including cold seeps and hydrothermal vents, using databases such as SciFinder, Web of Science, and so on. The structures of these compounds and details of the source organisms and depth of collection are presented along with relevant biological activities of the metabolites and synthetic studies. A total number of 182 compounds are presented in this review, with 132 cited references.

## 2. Cold Seeps

### 2.1. Marine Animals

Marine animals generally contain high proportions of *n*-3 polyunsaturated fatty acids (PUFAs) [7], in particular, long-chain PUFAs, such as DHA and EPA. There is increasing evidence that specific dietary patterns including, for example, *n*-3 PUFAs may be beneficial in reducing breast cancer risk [8,9]. However, some bivalve symbiotic bacteria were found to contain a novel *n*-4 or *n*-7 family, which appears to be an adaptation to the extremely high pressure and low temperature of seawater [10].

**Figure 1:**
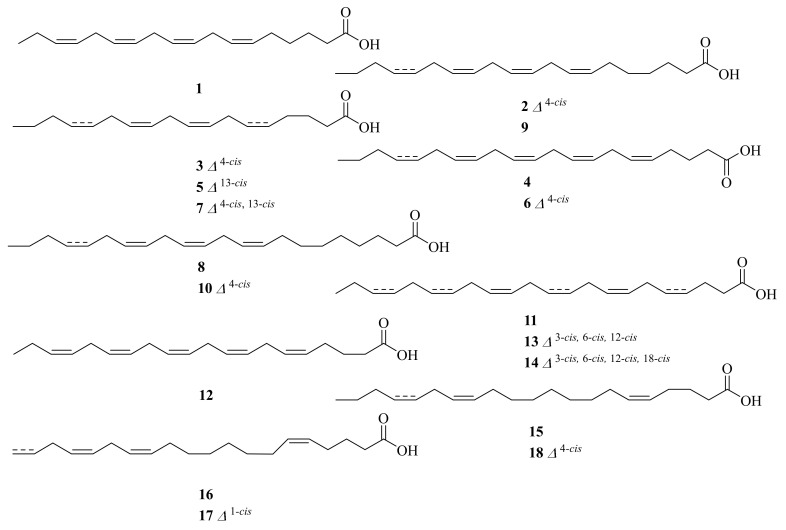
The chemical structures of compounds (**1**–**18**).

Novel fatty acids (**1**–**10**) (Figure 1) were purified from the two cold-seep-derived mussels *Bathymodiolus japonicus* and *B. platifrons*, collected at a depth of 1209 m at latitude 35°18′ N and longitude 139°13′ E in the Northern Pacific Ocean and a depth of 978 m at latitude 27°47′ N and longitude 126°54′ E in the East China Sea. The major PUFAs in the two mussels belong to unusual *n*-4 and *n*-7 methylene-interrupted PUFAs. *B. japonicus* and *B. platifrons* could maintain fluidity in plasma membrane lipids by accumulation of *n*-4 family methylene-interrupted PUFAs [11].

The cold-seep clam *Calyptogena phaseoliformis*, collected in the Japan Trench at a depth of 6354–6367 m, yielded eight novel fatty acids (**11**–**18**) (Figure 1). They were determined by gas chromatography–mass spectrometry analysis of 4,4-dimethyloxazoline derivatives. The major fatty acids present in *C. phaseoliformis* lipids belong to the *n*-4 family non-methylene-interrupted PUFAs [12].

**Figure 2:**
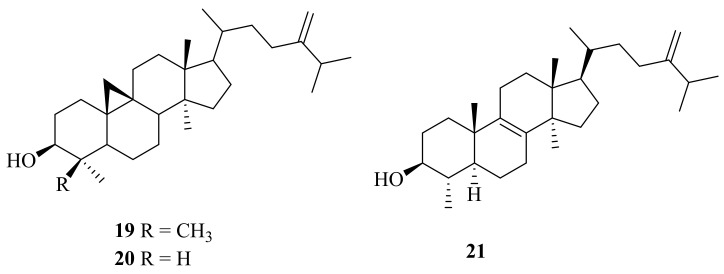
The chemical structures of compounds (**19**–**21**).

From the cold-seep bivalve *Calyptogena soyoae*, which was collected at a depth of 1100 m in Sagami Bay, three sterols (**19**–**21**) (Figure 2) were isolated [13]. Among them, 24-methylenecycloartanol (**19**) had shown many biological activities, such as significant anti-diabetic activity [14], strong activity against 12-*O*-tetradecanoylphorbol-13-acetate (TPA)-induced inflammation [15], and promising inhibition of growth of human breast cancer (MCF-7), with an IC_50_ value of 16.93 μM [16]. Cycloeucalenol (**20**) had been reported in the form of cycloeucalenol *trans*-ferulate in rice germ and was also found in *Tinospora cordifolia* or *Guduchi*. Its biological effects include cardiotonic [17], anti-fungal [18], and anti-inflammatory activities [15]. Compound **21** was evaluated for its cytotoxicity against MCF-7 and MDA-MB231, which clearly inhibited cell growth, with IC_50_ values of 29.33 ± 1.52 and 41.81 ± 2.42 μM, respectively [19].

### 2.2. Marine Fungi

#### 2.2.1. *Aspergillus* sp.

*Aspergillus* is one of the most common and important genera of fungus. It has attracted more and more scientists’ attention, with a variety of active secondary metabolites [20,21].

**Figure 3:**
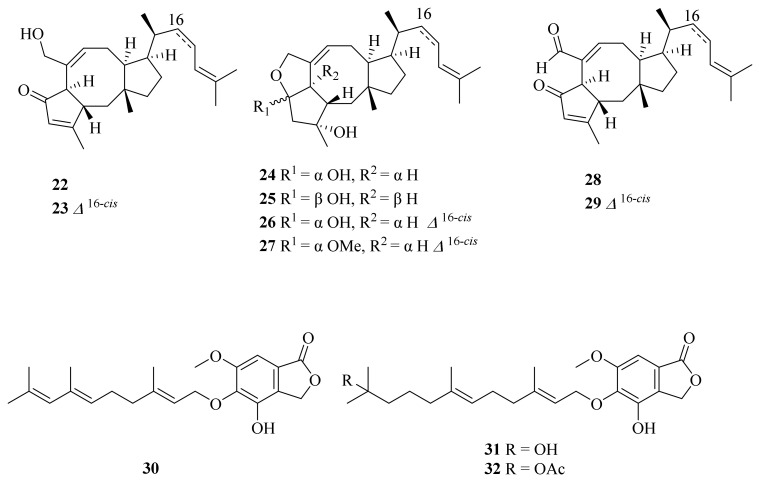
The chemical structures of compounds (**22**–**32**).

A deep-sea-derived fungus, *Aspergillus insuetus* SD-512, which was obtained from cold seep sediments collected at a depth of 1331 m, yielded three new ophiobolin sesterterpenoids (**22**–**24**) and three new farnesylated phthalide derivatives, farnesylemefuranones D–F (**30**–**32**), along with five known ophiobolin analogs (**25**–**29**) (Figure 3). Of them, compound **24** displayed broad-spectrum antibacterial activities with minimum inhibitory concentration (MIC) values ranging from 4 to 32 μg/mL [22]. Compound **26** was found to be active against *Escherichia coli*, with inhibitory diameters of 10 mm [23]. Compounds **25**–**27** were evaluated for cytotoxic activity against murine L5178Y lymphoma cells. However, none of them showed significant activity [24]. Compounds **28** and **29** were firstly isolated from extracts of *Emericella variecolor* GF10, which was separated from marine sediment [25]. Compound **28** showed potent cytotoxicity, with GI_50_ (growth inhibition) values ranging from 0.20 to 0.30 µM, against six cancer cell lines, HCT-15, NUGC-3, NCI-H23, ACHN, PC-3, and MDA-MB-231 [26]. In addition, the total synthesis of (−)-6-*epi*-ophiobolin N (**28**) was reported [27]. 6-*epi*-Ophiobolin G (**29**) exhibited potent cytotoxic activity against HepG2, with an IC_50_ value of 0.37 μM [28]. Compound **30** exhibited inhibitory effects against the aquatic pathogens *Vibrio vulnificus* QDIO-4 and *Vibrio alginolyticus* QDIO-7, with a MIC value of 4 μg/mL, while compound **32** showed further activity against the aquatic bacteria *Vibrio vulnificus* QDIO-4, *Vibrio alginolyticus* QDIO-7, and *Edwardsiella tarda* QDIO-8, with a MIC value of 4 μg/mL [22].

**Figure 4:**
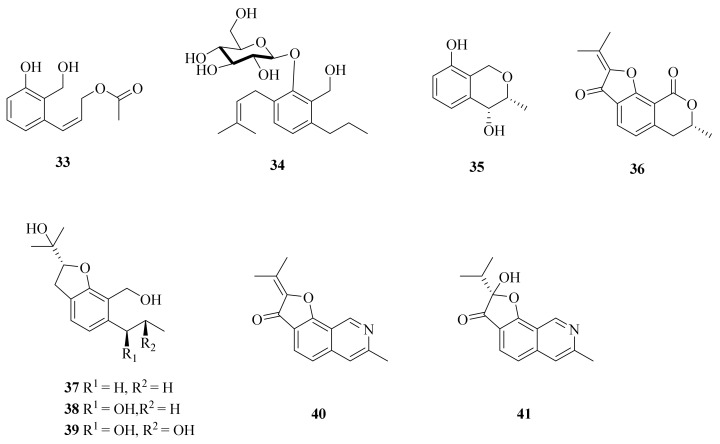
The chemical structures of compounds (**33**–**41**).

Moreover, *Aspergillus insuetus* SD-512 also yielded one new phenol derivative—acetylpeniciphenol (**33**)—along with eight known analogs (**34**–**41**) (Figure 4) [3]. Compound **33** was tested for antibacterial activities against six human or aquatic pathogens, while it exhibited an inhibitory effect against *Edwardsiella tarda*, *Vibrio alginolyticus*, and *V. vulnificus*, with MIC values of 4, 8, and 8 μg/mL, respectively [3]. Compound **34** displayed no significant activity in inhibiting LPS-induced NO production in RAW264.7 macrophages [29]. The biosynthetic pathway of penicisochroman E (**35**) was clarified; it involves epoxidation and cyclization followed by dehydration and subsequent hydrogenation [30]. (−)-Brassicadiol (**37**) exhibited cytotoxicity against both cancerous and non-cancerous (Vero) cells, with IC_50_ values ranging from 66.3 to 113.3 μM [31]. The synthesis of (−)-brassicadiol (**37**) was also described [32]. One study showed that daldinin C (**38**) was firstly isolated from cultures of the ascomycete *Daldinia concentrica* [33]. The anti-HIV activity of daldinin C (**38**) was tested, but the results were negative [34]. Penicisochroman I (**39**) showed weak cytotoxicity against KB and NCI-H187 cells [31]. TMC-120B (**40**) and TMC-120C (**41**) were observed to significantly lower PTZ-induced seizures in the larval zebrafish PTZ seizure model [35]. Because compound **41** has significant activity, its total synthesis route was also studied [36].

**Figure 5:**
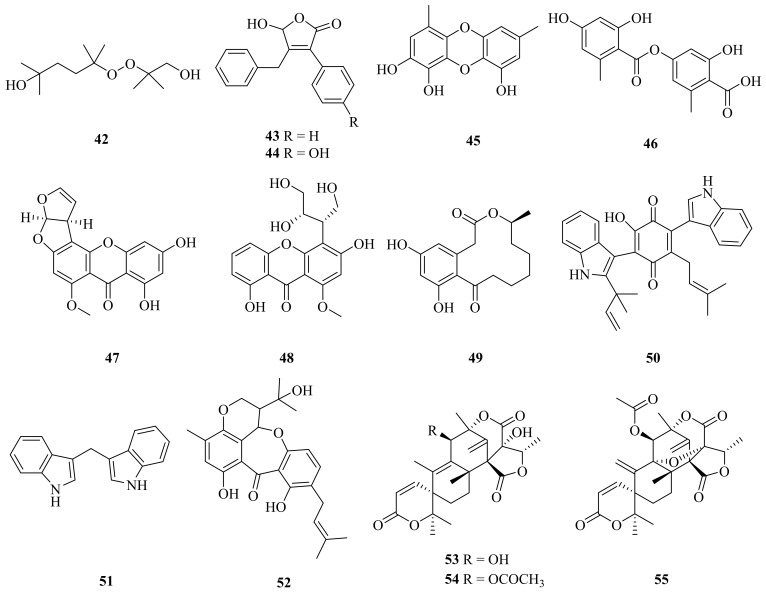
The chemical structures of compounds (**42**–**55**).

A new acyclic peroxide derivative, asperoxide A (**42**), and 13 known compounds (**43**–**55**) (Figure 5) were reported in 2020 from the deep-sea cold-seep species *Aspergillus nidulans* SD-531. All of the isolated compounds were tested for antimicrobial activities against human and aquatic bacteria as well as plant pathogenic fungi. Compounds **42**–**51** exhibited antimicrobial activities against some of the tested strains, with MIC values ranging from 2 to 64 μg/mL [37]. An improved synthesis of microperfuranone (**43**) (six steps, 56% yield) was reported [38]. 9-Hydroxymicroperfuranone (**44**) was also isolated from the fungus *Emericella quadrilineata* IFM42027 [39]. Compound **45** displayed the strongest antibacterial activities among the tested samples and may be a promising natural antimicrobial agent [37]. Lecanoric acid (**46**) exhibited potent free radical scavenging activity and showed significant Nrf2 activation [40]. Sterigmatocystin (**47**) displayed promising antibacterial activity, especially on *Pseudomonas aeruginosa*, with a MIC of 125 μg/mL [41]. Sterigmatocystin (**47**) also showed cytotoxic activities against HepG2, Hela, MCF-7, and HT-29, with IC_50_ values of 12.50 ± 0.89 μM, 11.50 ± 0.99 μM, 6.76 ± 0.31 μM, and 8.16 ± 0.39 μM, respectively [42]. Curvularin (**48**) was active against fungi and numerous cancer cell lines [43], and the total synthesis of curvularin (**48**) was achieved through a ring-closing-metathesis-based construction of the macrocyclic framework [44]. Terrequinone A (**50**) was found to be cytotoxic, with IC_50_ values ranging from 5.40 to 13.90 µM against four cancer cell lines (NCI-H460, MCF-7, SF-268, and MIA Pa Ca-2) and normal human primary fibroblast cells (WI-38) [45]. 3,3′-Diindolylmethane (**51)** showed many biological activities, such as extensive anticancer activity [46,47], adipogenesis properties [48], and an antioxidant function [49]. Compound **52** displayed acetylcholinesterase (AchE) inhibitory activity, with an IC_50_ value of 0.40 µM [50]. Compound **53** was evaluated for its cytotoxicity toward HTB-176 human lymphoma cells, with an IC_50_ of 10 ± 3.92 μM. Compound **53** also demonstrated significant antibacterial activity against *P. aeruginosa* [51]. In addition, one study reported the mechanistic details of the enzyme-catalyzed, stereospecific spiro-lactone ring-forming reaction to produce austinol (**53**) [52]. Compound **54** exhibited considerable cytotoxicity against HL-60 and SU-DHL-4 tumor cell lines, with IC_50_ values of 18.9 and 25.6 μM, respectively [53]. Compounds **53**–**55** also exhibited potent neuraminidase inhibitory activity [54].

#### 2.2.2. *Penicillium* sp.

*Penicillium* fungi have received remarkable interest as an important source of novel natural products encompassing diverse chemical structures and bioactive properties [55,56].

**Figure 6:**
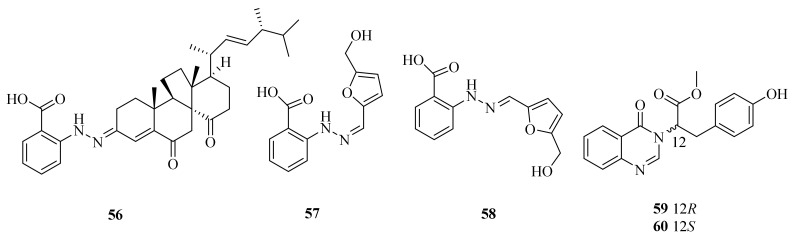
The chemical structures of compounds (**56**–**60**).

The fungus *Penicillium oxalicum*, obtained from a deep-sea cold seep, was found to produce three new phenylhydrazones, penoxahydrazones A–C (**56**–**58**), and two new quinazolines, penoxazolones A (**59**) and B (**60**) (Figure 6). Compounds **56**, **59**, and **60** could inhibit *Chattonella marina*, *Heterosigma akashiwo*, and *Prorocentrum donghaiense*, with IC_50_ values ranging from 0.57 to 9.1 µg/mL. Isolates **56**, **59**, and **60** also showed moderate inhibition against *V. harveyi* and *V. parahaemolyticus*, with inhibition zone diameters exceeding 10 mm at 20 µg/dis [57].

#### 2.2.3. *Cladosporium* sp.

Marine-associated *Cladosporium* species have attracted considerable interest because of their ability to produce a wide array of metabolites, including alkaloids, macrolides, diketopiperazines, pyrones, tetralones, sterols, phenolics, terpenes, and lactones, that possess versatile bioactivities [58,59].

**Figure 7:**
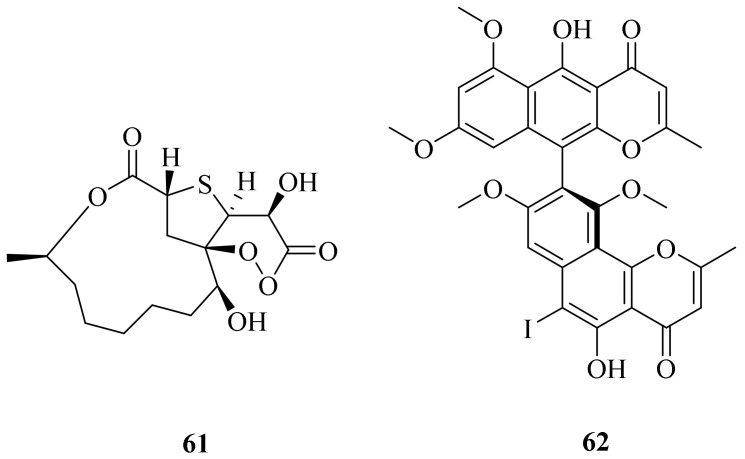
The chemical structures of compounds (**61**–**62**).

Cladosporioidin A (**61**), which possesses a novel sulfur and peroxy-bridged twelve-membered macrolide, and a new iodinated dimeric naphtho-γ-pyrone, (a*S*)-6-iodofonsecinone A (**62**) (Figure 7), were obtained from a cold seep isolate (8–1) of *Cladosporium cladosporioides*. Compound **61** was found to exhibit weak antibacterial ability against three bacteria (*Vibrio harveyi*, *V. anguillarum*, and *Pseudoalteromonas citrea*), with inhibitory zone diameters of 7.0, 7.0, and 8.0 mm, respectively. Compound **62** appeared to be the most potent against *P. citrea*, with an IC_50_ value of 0.61 μg/mL [60].

#### 2.2.4. *Curvularia* sp.

Secondary metabolites of the genus *Curvularia* revealed fascinating biological activities, including anti-malarial, anti-biofouling, anti-larval, and anti-inflammatory activities [61].

**Figure 8:**
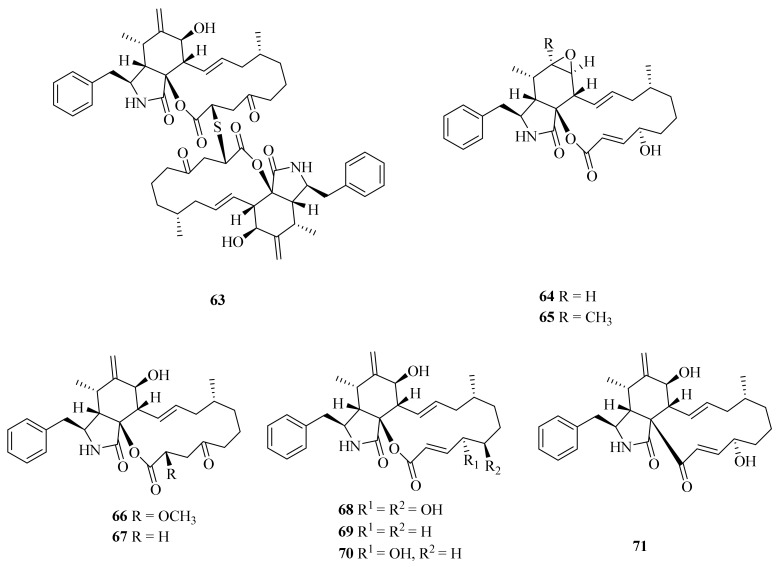
The chemical structures of compounds (**63**–**71**).

The deep-sea cold-seep endozoic fungus *Curvularia verruculosa* CS-129, retrieved from an area in the South China Sea, has yielded a new cytochalasin dimer—verruculoid A (**63**)—three new cytochalasin derivatives (**64**, **66**, and **68**), and a synthetic product obtained as a natural product for the first time (**69**) together with four known analogs (**65**, **67**, **70**, and **71**) (Figure 8). Compound **63** displayed activity against the human pathogenic bacterium *Escherichia coli* (MIC = 2 μg/mL) [62]. Cytochalasin B (**65**) had the best effect on the actin cytoskeleton [63]. Cytochalasin B_6_ (**67**) was firstly isolated from a jellyfish-derived fungus, *Phoma* sp., and showed moderate cytotoxicity [64]. Compounds **68**, **70**, and **71** showed cytotoxicity against HCT-116, HepG-2, and MCF-7, with IC_50_ values from 5.2 to 12 μM [62]. Deoxaphomin (**71**) also exerted the most marked inhibitory effects on the growth of six cancer cell lines: the human OE21 esophageal, U373 glioblastoma, SKMEL28 melanoma, A549 non-small cell lung cancer, mouse B16F10 melanoma, and human HS683 oligodendroglioma cell lines [65].

### 2.3. Marine Bacteria

#### 2.3.1. *Streptomyces* sp.

*Streptomyces* sp. Have well-developed branching hyphae, and more than 1000 species have been reported, mainly distributed in soil. They are attractive microbial cell factories that have industrial capabilities to produce a wide array of bioactive secondary metabolites [66,67].

**Figure 9:**
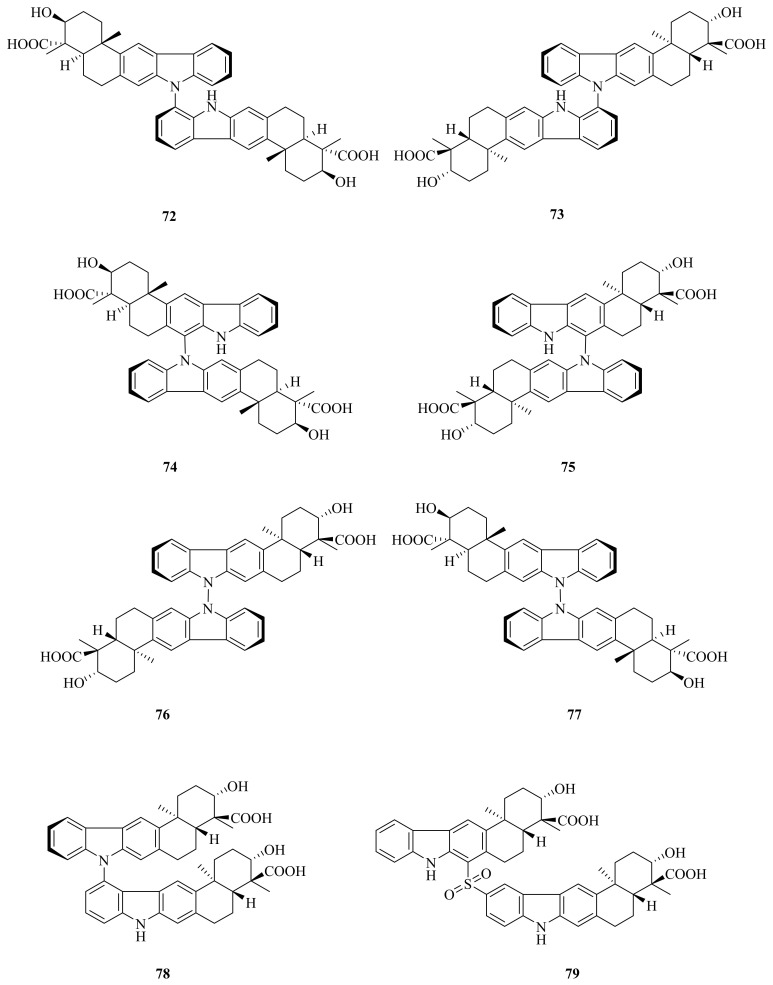
The chemical structures of compounds (**72**–**79**).

A cold-seep-derived actinomycete belonging to the *Streptomyces olivaceus* OUCLQ19-3 genus was found to contain two new (**72** and **73**) and six known (**74**–**79**) (Figure 9) dixiamycins. In the antibacterial test, compounds **72**–**79** exhibited significant growth inhibition against several multi-drug-resistant (MDR) strains, with MIC values ranging from 0.78 to 6.25 μg/mL; among these, **72**, **73**, and **76**–**79** were more potent than the positive control tetracycline [4]. Dixiamycins A (**77**) and B (**76**) are the first examples of atropisomerism naturally occurring in N–N-coupled atropo-diastereomers [68]. A unique method of electrochemical dimerization of carbazoles and carbolines enabled the first total synthesis of dixiamycin B (**76**) [69]. Sulfadixiamycin A (**79**) was found to have selective yet moderate antimycobacterial properties, with a MIC value of 25 mg/mL [70].

#### 2.3.2. *Halomonas* sp.

*Halomonas* is a kind of Gram-negative bacterium which has strong adaptability and a wide range of adaptability to temperature, salinity, and oxygen. It may have important application values in sewage treatment and bioremediation [71].

An immune-enhancing exopolysaccharide, EPS2E1 (**80**), was reported in 2021 from a cold-seep bacterium, *Halomonas* sp. 2E1, which was collected in the South China Sea (119°17′ 04.956″ E, 22°06′58.384″ N; 1142 m deep). Structural analysis showed that the backbone mainly consisted of →2)-Man-(α-1→ and →2, 6)-Man-(α-1→ in a ratio of 2.45:1.00. The chain contained →4)-Glc-(α-1→, →6)-Man-(α-1→and→3)-Glc-(β-1→). EPS2E1 exhibits the potential to be an immunopotentiator, because it could significantly increase the production of NO, COX-2, TNF-α, IL-1β, and IL-6 by activating the MAPK and NF-κB pathways on RAW264.7 macrophages. [62,72].

#### 2.3.3. *Vibrio* sp.

Bacteria belonging to the *Vibrio* family are short in shape and named for their curve-like arcs. They are usually found in freshwater or seawater and also in the intestines of humans or fish. Some species are pathogenic to fish or humans [73]. *Vibrio* species can produce compounds with attractive biological activities, including antibacterial, anticancer, and antivirulence activities [74].

In 2021, the isolation of a novel exopolysaccharide, EPS364 (**81**), was reported from a deep-sea cold-seep fungus, *Vibrio alginolyticus* 364, obtained in the South China Sea (119°17′05.3940″ E, 22°06′58.7264″ N). EPS364 consisted of mannose, glucosamine, gluconic acid, galactosamine, and arabinose in a molar ratio of 5:9:3.4:0.5:0.8. Notably, EPS364 exhibited a significant antitumor activity, inducing apoptosis, dissipation of the mitochondrial membrane potential (MMP), and generation of reactive oxygen species (ROS) in Huh7.5 liver cancer cells, which suggests that EPS364 is a promising antitumor agent for pharmacotherapy [75].

#### 2.3.4. *Bacillus* sp.

Marine *Bacillus* species produce versatile secondary metabolites, including lipopeptides, polypeptides, fatty acids, polyketides, and coumarins. These structurally diverse compounds exhibit a wide range of biological activities [76].

A bacterial strain isolated from the cold-seep-derived fungus *Bacillus* sp. CS30 which was collected in the South China Sea in October 2017 (119°17′09.655″ E, 22°06′5.169″ N), exhibited strong growth inhibition against *M. grisea*. Two purified antifungal agents were isolated which belong to the surfactin family and were named surfactin CS30-1 and surfactin CS30-2 (**82** and **83**). Both of them showed antifungal activity, since they could induce the generation of reactive oxygen species (ROS) and caused serious damage to the cell wall and cytoplasm [77].

### 2.4. Others

**Figure 10:**
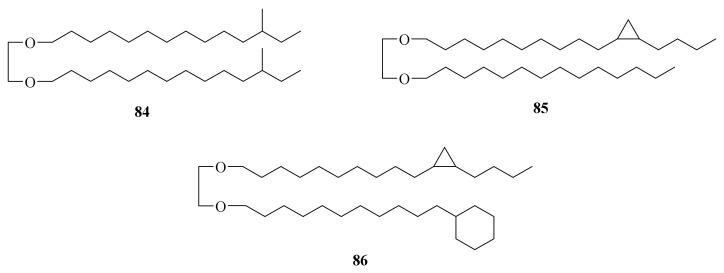
The chemical structures of compounds (**84**–**86**).

Three novel series of non-isoprenoidal dialkyl glycerol diethers were tentatively identified in carbonate crusts precipitated from methane-rich bottom-waters and pore-waters associated with Mediterranean mud volcanoes (**84**–**86**) (Figure 10). All of the reported sedimentary compounds represent the first detailed report on the occurrence of alkyl diethers in a non-thermophilic setting, and the cyclopropyl and cyclohexyl moieties as observed in the series I and II components are unique for ether lipids [78].

## 3. Hydrothermal Vents

### 3.1. Marine Animal

**Figure 11:**
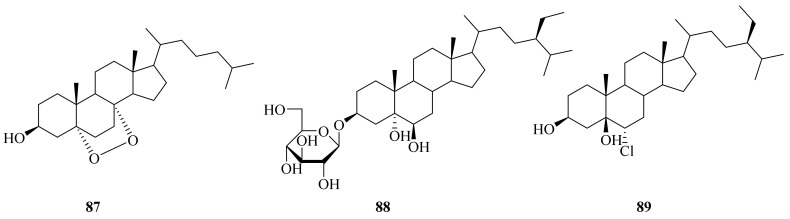
The chemical structures of compounds (**87**–**89**).

Three sterols were isolated (**87**–**89**) (Figure 11) from the species of bivalve *Bathymodiolus septemdierum*, which was collected in 2004 at a depth of 1244 m from hydrothermal vents at Myojin Knoll, Japan. Their unique feeding modes and metabolism of nutrients make the structures of their natural products more novel [13]. Compound **88** showed allelopathic activity against *Lactuca sativa* seedlings and autotoxic activity against *A. hoantchy* seedlings [79]. The total synthesis of 5α, 6β-dihydroxystigmastan-3-*O*-β-glycopyranoside (**89**) was reported [80].

### 3.2. Marine Fungi

#### 3.2.1. *Penicillium* sp.

**Figure 12:**
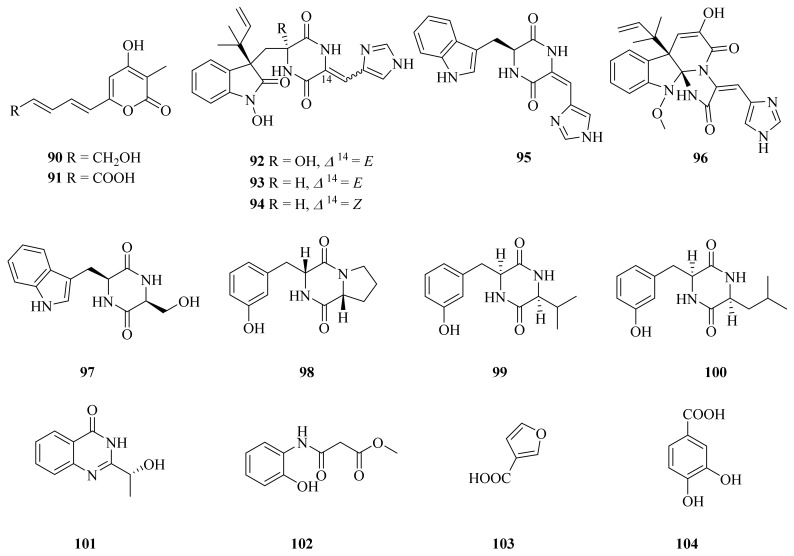
The chemical structures of compounds (**90**–**104**).

In 2020, Han et al. described the isolation of three new compounds (**90**–**92**) along with twelve known compounds (**93**–**104**) (Figure 12) from a deep-sea hydrothermal fungus, *Penicillium chrysogenum* SCSIO 07007, collected from the Western Atlantic (126.8983° E, 27.7875° N) at a depth of 1028 m. Of them, chrysopyrones A and B (**90** and **91**) showed obvious inhibitory activities against protein tyrosine phosphatase 1B (PTP1B), with IC_50_ values of 9.32 and 27.8 μg/mL, respectively [81]. Meleagrin (**96**) exhibited a variety of activities, such as antitumor [82], cytotoxic [83], antibiofilm, and antifouling activities [84]. Cyclo (Trp-Ser) (**97**) displayed antibacterial activity against *Escherichia coli*, *Chromobacterium violaceum* CV026, *Pseudomonas aeruginosa* PA01, *Staphylococcus aureus*, and *Candida albicans*, with MIC values ranging from 3.2 to 6.4 mg/mL [85]. Cyclo (Pro-Tyr) (**98**) exhibited weak antibacterial activity against *X. axonopodis pv. citri* and *R. solanacearum* but showed a MIC of 31.25 μg/mL [86]. The biosynthesis of chrysogine (**100**) was proven to be related to a candidate NRPS cluster comprising five additional genes named *chry*2–6 gene clusters [87]. 2-Furoic acid (**103**) was shown to be effective in lowering both serum cholesterol and serum triglyceride levels, significantly in rats with an elevation of HDL cholesterol levels at 20 mg/kg/day orally [88]. 3,4-Dihydroxybenzoic acid (**104**) may be an important phenolic compound in regulating root formation in *P. cynaroides* cuttings [89].

**Figure 13:**
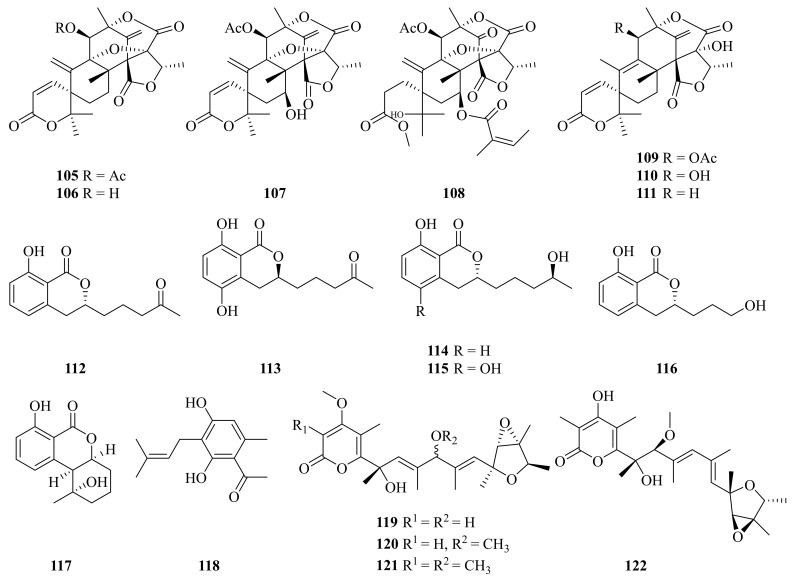
The chemical structures of compounds (**105**–**122**).

Five new compounds (**108**, **113**, **115**–**117**) together with eight known compounds (**105**–**107**, **109**–**112**, **114**, and **118**) (Figure 13) were obtained from *Penicillium* sp. Y-5-2, which was collected in May 2014 from Kueishantao, off Taiwan. New compounds **113**, **115**, and **117** revealed inhibitory activities against *E. coli* at MIC values around 32 μg/mL [90]. Dehydroaustin (**105**) was an attractive natural insecticide with a LC_50_ value of 2.9 ppm [91]. Compounds **105** and **106** showed acetylcholinesterase (AchE) inhibitory activity, with IC_50_ values of 0.40 and 3.00 μM, respectively [50]. Dehydroaustinol (**106**) and austin (**109**) displayed considerable cytotoxicity against the HL-60 and SU-DHL-4 tumor cell lines, with IC_50_ values ranging from 18.9 to 27.8 μM [53]. Austinol (**110**) exhibited strong antibacterial activity against the *P. aeruginosa* bacterial strain, with a MIC value of 0.13 ± 0.4 µg/mL [51]. Aspergillumarins A (**112**) and B (**114**) showed weak antibacterial activity against *Staphylococcus aureus* and *Bacillus subtilis* at a concentration of 50 μg/mL [92]. Pestalotionol (**118**) showed potent antibiotic activity against *Staphylococcus aureus* and *Bacillus subtilis*, with MIC values of 8 and 2 μg/mL, respectively [90]. Compound **118** also showed weak anti-inflammatory activity by measuring the nitric oxide (NO) production in lipopolysaccharide (LPS)-activated RAW264.7 macrophages [93].

In 2020, Pan and colleagues isolated four verrucosidin derivatives (**119**–**122**) (Figure 13) from the sulfur-derived fungus *Penicillium* sp. Y-50-10, collected in the Kueishantao hydrothermal vents off Taiwan [94]. Compounds **119**–**122** showed activity against *Bacillus subtilis*, with MIC values of 32 μg/mL [95].

#### 3.2.2. *Aspergillus* sp.

**Figure 14:**
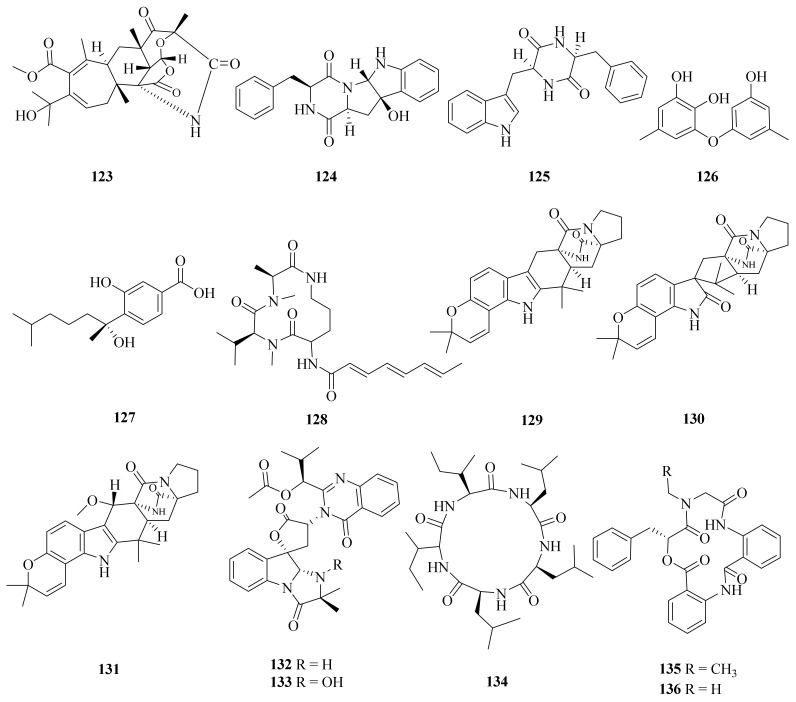
The chemical structures of compounds (**123**–**136**).

In 2016, the strain *Aspergillus* sp. WU 243, collected from the digestive gland of *Xenograpsus testudinatus*, a unique type of crab which dwells in the Kueishantao hydrothermal vents off Taiwan, was reported to contain a novel hybrid polyketide-terpenoid, aspergstressin (**123**), and four known compounds (**124**–**127**) (Figure 14) [96]. Cyclo-(Try-Phe) (**125**) can be used as a plant growth regulator; it exhibited different biological activities against the tested plants [97]. Cordyol C (**126**) exhibited significant anti-HSV-1 activity, with an IC_50_ value of 1.3 μg/mL, and cytotoxic activity against BC and NCI-H187 cancer cell lines, with IC_50_ values of 8.65 and 3.72 μg/mL, respectively [98]. Cordyol C (**126**) was also a toxic compound against HeLa cells, with an IC_50_ value of 35.29 ± 1.55 mM [99]. Sydowic acid (**127**) was assessed in murine leukemia P-388 cells and showed potential cytotoxicity, with an IC_50_ value of 20.30 μg/mL [100].

Four secondary metabolites (**128**–**131**) (Figure 14) were isolated from the hydrothermal fungus *Aspergillus sclerotiorum* C10WU, which was collected from Kueishantao, Taiwan. Stress metabolite **128** was reported to possess insecticidal activities and show cytotoxic effects against human cervical carcinoma [101]. Stephacidin A (**129**) is proposed as a biosynthetic precursor to notoamide B in various *Aspergillus* species. Following a strategy based on doubly ^13^C-labeled stephacidin A (**129**), it could undergo bio-transformation to notoamide B (**130**) [102]. In addition, the total synthesis of the natural indole alkaloid notoamide F (**131**) was reported [103].

**Figure 15:**
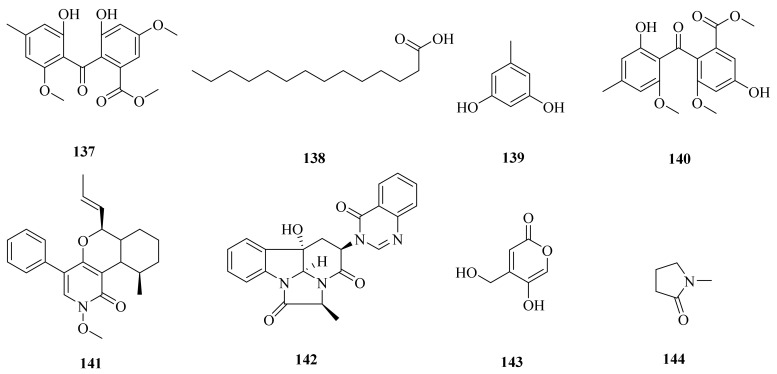
The chemical structures of compounds (**137**–**144**).

A hydrothermal fungus Aspergillus clavatus C2WU, which was also collected from Kueishantao, Taiwan, yielded two secondary metabolites (**132** and **133**) (Figure 14). Notably, deoxytryptoquivaline (**132**) showed strong binding to three targets, SARS-CoV-2 main protease and spike glycoprotein and human angiotensin-converting enzyme 2. Therefore, it has promise for being further investigated as a possible multitarget drug against COVID-19 [104]. Aspergillus clavatus C2WU also yielded a unique new cyclopeptide, clavatustide C (**134**) (Figure 15), which was produced as a stress metabolite in response to abiotic stress elicitation by one of the hydrothermal vent’s fluid components, Zn [105]. Moreover, two novel cyclodepsipeptides, namely, clavatustides A (**135**) and B (**136**) (Figure 15), were also purified from Aspergillus clavatus C2WU. Clavatustides A (**135**) and B (**136**) displayed antitumor activity by suppressing the proliferation of hepatocellular carcinoma (HCC) cell lines (HepG2, SMMC-7721, and BEL-7402), inducing an accumulation of HepG2 cells in G1 phase and a reduction in cells in S phase [106]. The enantiopure synthesis of clavatustides A (**135**) and B (**136**) was accomplished by a seven-step synthetic protocol starting from commercially available (R)-phenyllactic acid [107].

One new compound (**137**) and seven known compounds (**138**–**144**) (Figure 15) were obtained from *Aspergillus* sp. YQ-13, collected from the sediment of Kueishantao hydrothermal vents off Taiwan [108]. Notably, myristic acid (**138**) showed various biological activities, for example, specifically blocking T cell antigen receptor CD_3_-induced Ca^2+^ mobilization in T cells [109]; exhibiting antibacterial activity [110]; and reducing type 2 diabetes risk [111]. Orcinol (**139**) exhibited remarkable antioxidant activity; its free radical scavenging rate can reach up to 80% of 20 mg/mL [112]. Compounds **137** and **139** were tested by the methods of DPPH and FRAP assays, showing moderate antioxidant activities [108]. 1,2-*seco*-Trypacidin (**140**) exhibited a weak inhibitory effect on *Helicobacter pylori* 159, with a MIC of 16 μg/mL [113]. Leporin A (**141**) and chaetominine (**142**) exhibited antibiotic activity, with MIC values around 1 to 25 μg/mL against *Bacillus subtilis*, *Klebsiella pneumoniae*, methicillin-resistant *Staphylococcus aureus* (MRSA), *Pseudomonas aeruginosa*, *Staphylococcus aureus*, *Escherichia coli*, and *Acinetobacter Bauman* [108]. 4-(Hydroxymethyl)-5-hydroxy-2H-pyran-2-one (**143**) induced the production of cAMP in a dose-dependent manner, which indicated that **143** might be a possible ligand of GPR12 [114]. Compound **143** also has significant antioxidant activity, with an IC_50_ value of 59.5 µM [115], and weak inhibition of bacterial growth [116].

**Figure 16:**
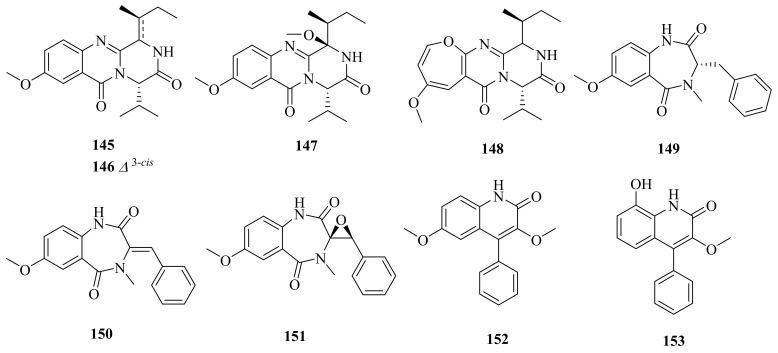
The chemical structures of compounds (**145**–**153**).

Three new quinazoline derivatives (**145**–**147**), one new oxepine-containing natural product (**148**), four new cyclopenin derivatives (**149**–**151** and **153**), and one known compound (**152**) (Figure 16) were isolated from an ethyl acetate extract of a hydrothermal vent crab belonging to the genus *Aspergillus versicolor* XZ-4, collected from the Taiwan Kueishantao. Compounds **149** and **151**–**153** revealed inhibitory activities against *E. coli* at MIC values around 32 μg/mL [117]. 3,6-*O*-Dimethylviridicatin (**152**) was firstly isolated from the deep-sea-derived fungus *Aspergillus versicolor* SCSIO 05879 [118].

#### 3.2.3. *Graphostroma* sp.

**Figure 17:**
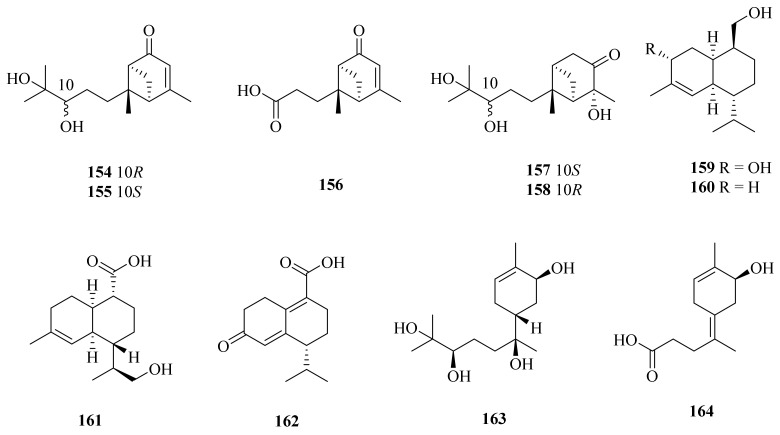
The chemical structures of compounds (**154**–**164**).

In 2017, the fungus *Graphostroma* sp. MCCC 3A00421, collected from a deep-sea hydrothermal sulfide deposit of the Atlantic Ocean (13.36° W, 15.17° S, at a depth of −2721 m), was reported to contain 11 sesquiterpene compounds (**154**–**164**) (Figure 17). Two of them are structurally connected (**154** and **155**), and nine are new compounds (**156**–**164**) [119]. Among them, compounds **154** and **155** were evaluated for their anticancer activity but had no significant effect against HL-60, A-549, MCF-7, SMMC-7721, and SW-480 human cancer cell lines [120]. Khusinol B (**159**) showed more significant anti-inflammatory activity than the positive control (aminoguanidine), with an IC_50_ value of 17 µM. In addition, compound **159** also showed weak anti-allergic activity, with an IC_50_ value of 150 µM [119].

### 3.3. Marine Bacteria

#### 3.3.1. *Streptomyces* sp.

**Figure 18:**
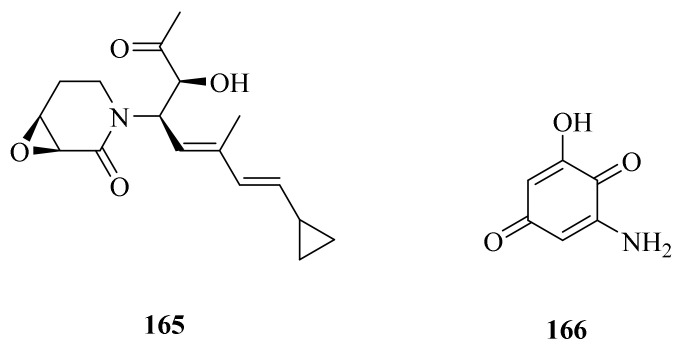
The chemical structures of compounds (**165**–**166**).

Hydrothermal vent microorganisms have a unique metabolic mechanism, because they have to withstand and respond to heavy metal concentrations [121]. A novel antibiotic (**165**) (Figure 18) was produced by *Streptomyces* sp. WU20, which was isolated from the metal-rich hydrothermal vents in Kueishantao, Taiwan. Compound **165** exhibited antimicrobial activity against *Bacillus subtilis*, with a MIC of around 32 μg/mL [122].

#### 3.3.2. *Geobacillus* sp.

*Geobacillus* is a Gram-positive bacterium, rod-shaped, and either paired or chained, and its optimum growth temperature is 65–70 degrees [123].

In 2017, the bacterium *Geobacillus* sp. E263, collected from a deep-sea hydrothermal vent in the East Pacific, was reported to contain a novel quinoid compound (**166**) (Figure 18). The research indicated that 2-amino-6-hydroxy-[1,4]-benzoquinone (**166**) could trigger the apoptosis of gastric cancer cells and breast cancer cells by inducing the accumulation of intracellular reactive oxygen species [124].

#### 3.3.3. *Halomonas* sp.

**Figure 19:**
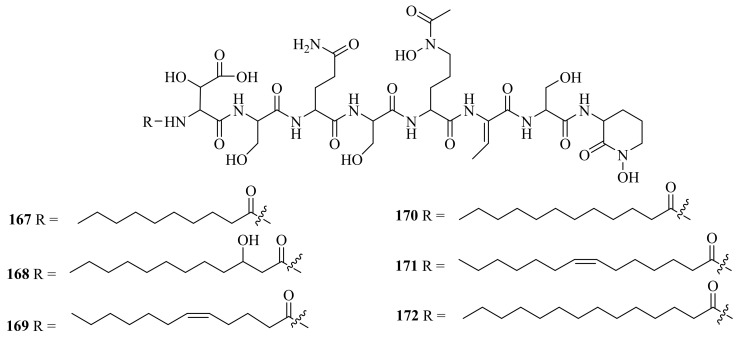
The chemical structures of compounds (**167**–**172**).

Six new amphiphilic siderophores, loihichelins A-F (**167**–**172**) (Figure 19), were obtained from cultures of the deep-sea hydrothermal vent and sulfide rock bacterium *Halomonas* sp. LOB-5, which was collected from Marker 17 (depth of 1714 m) at Loihi Seamount. These siderophores showed a potential role in the promotion of Mn(II) and Fe(II) oxidation [125]. In addition, the reports on loihichelins A-F were the first publications on new natural products from ocean hydrothermal vent environments.

#### 3.3.4. *Vibrio* sp.

An exopolysaccharide was produced under laboratory conditions by *Vibrio diabolicus*, a bacterium retrieved from a deep-sea hydrothermal vent in the East Pacific Rise (12°48.13′ N, 103°56.30′ W) (**173**). Structural analysis showed that the polysaccharide consists of a linear tetrasaccharide repeating unit with the following structure: →3)-β-D-Glcp NAc-(1→4)-β-D-GlcpA-(1→4)-β-D-GlcpA-(1→4)-α-D-Galp Nac-(1→ [126].

**Figure 20:**
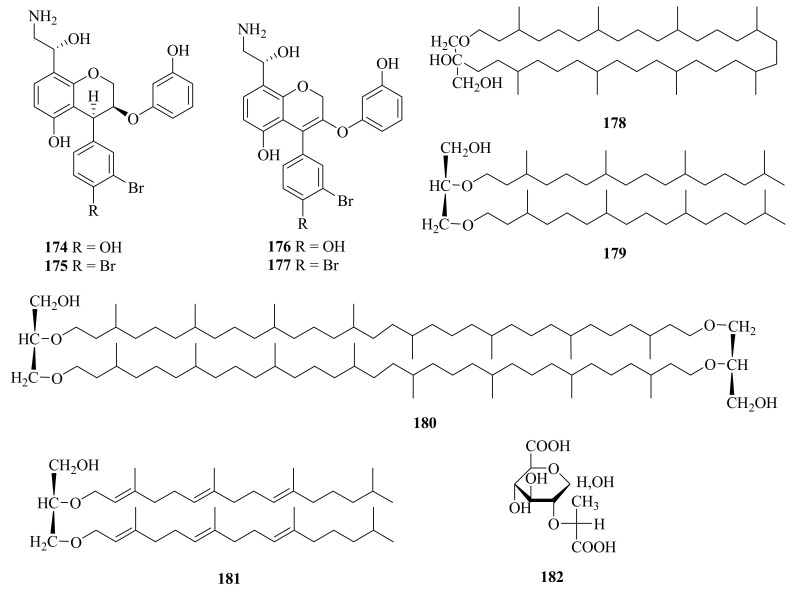
The chemical structures of compounds (**174**–**182**).

The bacterium *Thermovibrio ammonifican*, collected from a culture from marine hydrothermal vents in the East Pacific Rise (9°50′ N, 104°189′ W) at a depth of 2500 m, was found to contain four hydroxyethyl amine chromene derivatives, ammonificins A-D (**174**–**177**) (Figure 20) [127]. Ammonificins C (**174**) and D (**175**) could induce apoptosis at 2 μM and 3 μM, respectively (the control, staurosporine at 0.1 μM) [128].

#### 3.3.5. *Methanococcus* sp.

The membrane lipid of a new deep-sea hydrothermal vent methanogen, *Methanococcus jannaschii*, was isolated, purified, and structurally characterized (**178**) (Figure 20) [129].

#### 3.3.6. *Thermococcus* sp.

Three compounds (**179**–**181**) were isolated from the lipids of a deep-sea hydrothermal vent Archaeon, *Thermococcus* S557 (Figure 20). Among them, 2,3-di-*O*-dihydro-14,15-geranylgeranyl glycerol (**180**) is very likely a close intermediate in the biosynthesis of diphytanyl glycerol diether in Archaea [130].

#### 3.3.7. *Alteromonas* sp.

The exopolysaccharide produced by the bacterium *Alteromonas* sp. strain 1644 originating from deep-sea hydrothermal vents was shown to contain a novel glucuronic acid derivative: 3-*O*-[(*R*)-1-carboxyethyl]-*D*-glucuronic acid (**182**) (Figure 20) [131].

## 4. Comprehensive Overview and Outlook

**Figure 21:**
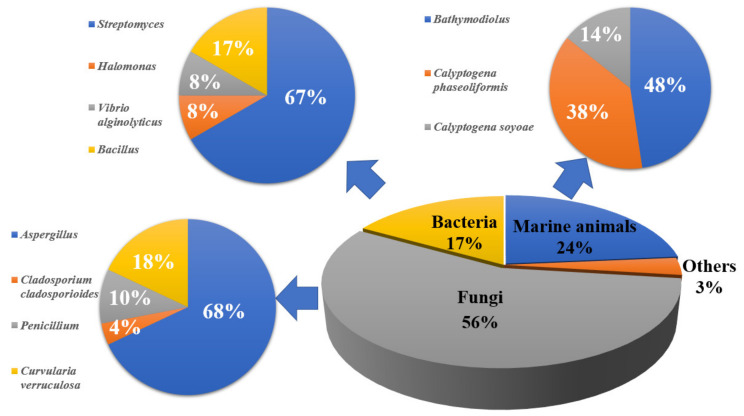
The sources of reported natural products from cold seeps.

**Figure 22:**
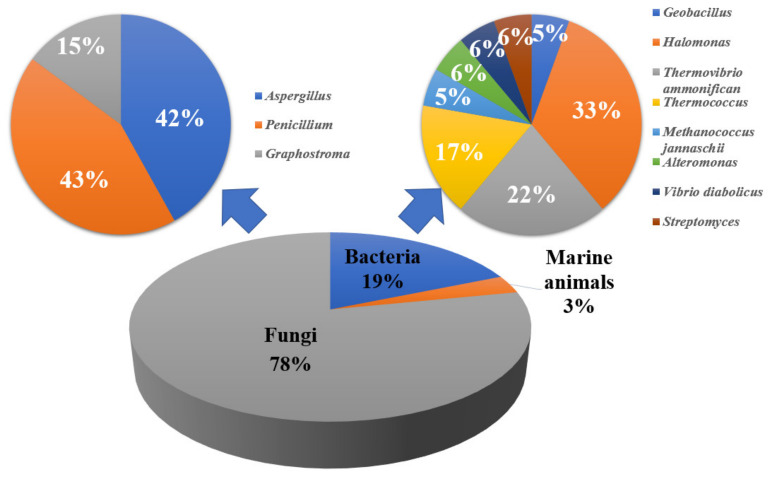
The sources of reported natural products from hydrothermal vents.

We provide a comprehensive overview of the sources and bioactivities of the 182 natural products from the deep-sea extreme environments described up to March 2022. It was observed that cold-seep-derived compounds could be divided into four parts, namely, marine animals (24%), fungi (56%), bacteria (17%), and others (3%). In general, they mainly come from *Aspergillus*, *Bathymodiolus*, and *Curvularia*, according to the number of compounds (Figure 21), suggesting that these genera would be subjected to the focus of future research. The secondary metabolites isolated from hydrothermal vents are found in three parts. At the domain level, 78% of the natural products were derived from fungi, while 19% originated from bacteria, among which *Aspergillus* and *Penicillium* were the main source of natural products (Figure 22).

**Figure 23:**
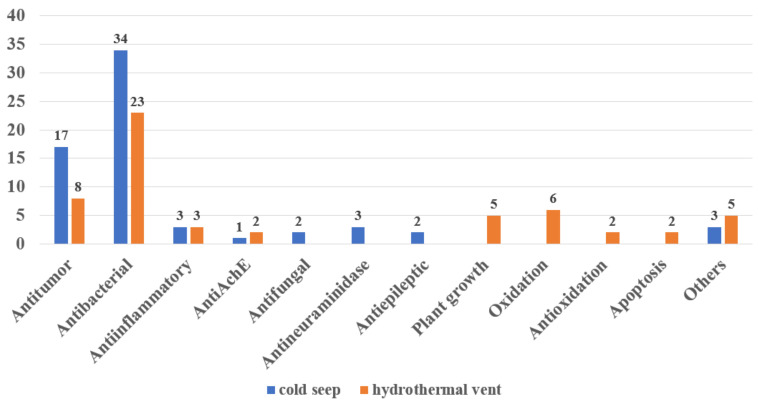
The bioactivities of the natural products from cold seeps and hydrothermal vents.

By comparing and analyzing the activities of secondary metabolites derived from cold seeps and hydrothermal vents, it was found that almost 60% of the 182 compounds had biological activities, and their activities were diverse (Figure 23). Among them, antibacterial and antitumor activities are reported most frequently. Some cold-seep-derived compounds also have antifungal and anti-epileptic activities, while hydrothermal vent-derived natural products also include plant growth regulation and oxidant activities. In general, that secondary metabolites derived from cold seeps and hydrothermal vents have novel and diverse biological activities may be due to their extreme and special environments.

## 5. Conclusions

There were 86 natural products isolated from cold seeps, while 96 secondary metabolites were isolated from hydrothermal vents. The sources of the compounds are represented by the genera *Aspergillus* sp., *Penicillium* sp., and so on. There are 90 new compounds among the 182 compounds. Around 60% of the deep-sea natural products were reported to possess bioactivity. For example, an exopolysaccharide, EPS364 (**81**), from cold-seep *Vibrio alginolyticus* 364, was investigated for its mechanism of inhibiting the growth and adhesion of liver cancer cells, which has proved to be the basis for a promising anticancer drug [75]. A hydrothermal vent-derived compound, deoxytryptoquivaline (**142**), showed strong binding to three important targets of SARS-CoV-2 and so has promise for being further investigated as a possible multitarget drug against COVID-19 [104]. These novel and diverse activities indicate that deep-sea extreme environments might facilitate the production of functional natural products. Moreover, the total synthesis or biosynthesis of some compounds was described. For example, the total synthesis pathway of (−)-6-*epi*-ophiobolin N (**28**), which was isolated from cold-seep sediments, was reported [27]. Dixiamycins A (**77**) and B (**76**), which were separated from a cold-seep environment sample, were reported in an unusual oxidative cyclization strategy for tailoring indolosesquiterpene biosynthesis [132] and in a possible route for total synthesis [69], respectively. These synthesized compounds either have a wide range of sources, diverse activities, or unique molecular skeletons rarely discovered in nature. This further indicates that the natural products derived from extreme environments, such as cold seeps and hydrothermal vents, have great potential and are a treasure to be further developed.
